# Serum C-reactive protein is a useful marker to exclude anastomotic leakage after colorectal surgery

**DOI:** 10.1038/s41598-020-58780-3

**Published:** 2020-02-03

**Authors:** Bruno A. Messias, Ricardo V. Botelho, Sarhan S. Saad, Erica R. Mocchetti, Karine C. Turke, Jaques Waisberg

**Affiliations:** 1 Department of Surgery, General Hospital of Carapicuiba, Carapicuiba, SP Brazil; 2 Department of Surgery, State Public Servant Hospital (IAMSPE), São Paulo, SP Brazil; 3 Department of Surgery, Paulista Medical School, Federal University of São Paulo, São Paulo, SP Brazil; 4 Department of Surgery, ABC Medical School, Santo André, SP Brazil

**Keywords:** Colonic diseases, Colorectal cancer

## Abstract

Anastomotic leakage is a complication of colorectal surgery. C-reactive protein (CRP) is an acute-phase marker that can indicate surgical complications. We determined whether serum CRP levels in patients who had undergone colorectal surgery can be used to exclude the presence of anastomotic leakage and allow safe early discharge. We included 90 patients who underwent colorectal surgery with primary anastomosis. Serum CRP levels were measured retrospectively on postoperative days (PODs) 1 – 7. Patients with anastomotic leakage (n = 11) were compared to those without leakage (n = 79). We statistically analysed data and plotted receiver operating characteristic curves. The incidence of anastomotic leakage was 12.2%. Diagnoses were made on PODs 3 – 24. The overall mortality rate was 3.3% (18.2% in the leakage group, 1.3% in the non-leakage group; *P* < 0.045). CRP levels were most accurate on POD 4, with a cutoff level of 180 mg/L, showing an area under the curve of 0.821 and a negative predictive value of 97.2%. Lower CRP levels after POD 2 and levels <180 mg/L on POD 4 may indicate the absence of anastomotic leakage and may allow safe discharge of patients who had undergone colorectal surgery with primary anastomosis.

## Introduction

Despite advances in surgical techniques and stapling devices, anastomotic leakage remains one of the most devastating complications of colorectal surgery^1,2^ with mortality rates reaching 30%^3^. The incidence of anastomotic leakage varies from 1% to 30%^4^, and it is most common in extraperitoneal anastomosis^2,5,6^. This variation in the incidence rate is attributed to the variety of definitions of anastomotic leakage found in the medical literature^3^.

Anastomotic leakage can be defined as a defect leading to the communication or extravasation of intra- and extraluminal contents^4,7^. The presence of a pelvic abscess or an abscess near the site of anastomosis can also be considered as leakage^5^. The most frequently reported risk factors for anastomotic leakage include male gender, smoking, obesity, preexisting disease (e.g., diabetes mellitus or chronic renal failure), nutritional status, use of neoadjuvant therapy, and emergency surgery^2,8–11^. Measures such as individualized hydration management, mechanical preparation of the colon, and use of epidural anaesthesia do not appear to influence the risk of leakage^11,12^.

Early diagnosis is essential to reduce mortality, length of hospital stay, postoperative complications, tumour recurrence, and costs^1,5^. Because of the high mortality rate^4^, scores were developed to help identify patients at high risk for this complication^13^. Clinical status may vary widely, however, from benign symptoms to signs of peritonitis and septic shock, and surgical intervention is frequently necessary^4,7,14,15^. Computed tomography (CT), endoscopic examination, biomarkers and abdominal drain secretion analysis are the most commonly used tools in clinical practice to diagnose anastomotic leakage^2,14,16,17^.

C-reactive protein (CRP) is the most widely studied biomarker^2,4,18,19^, first described in 1930 by Willian S. Tillet and Thomas Francis. It is considered an acute-phase protein^20–23^. Secretion begins at 4 to 10 hours following inflammatory stimulation, peaks in the plasma at 48 hours, and returns to baseline after the inflammatory stimulus ceases^20,21,24,25^. Because of its short half-life (19 hours), CRP is a reliable marker following surgical procedures^25–27^.

In addition to being used for the diagnosis of anastomotic leakage, CRP is also used as a marker of severity in gastrointestinal pathologies and infectious complications of open and laparoscopic surgeries^26,28–33^. The objective of this study was to determine whether serum CRP levels of patients who had undergone emergency or elective colorectal surgery with primary anastomosis can be used to exclude the presence of anastomotic leakage and allow for safe and early discharge.

## Methods

Ninety colorectal surgeries with primary anastomoses (ileocolic, colocolic, or colorectal) were performed in the General Surgery Department of Carapicuíba General Hospital between June 2014 and July 2018. The Research Ethics Committee of our institution approved this retrospective study and waived the need for informed consent. This study included patients of both gender who underwent elective or emergency colorectal surgery with primary anastomosis. The diseases that determined surgical treatment are presented in Table 1. Indications for emergency surgery included acute appendicitis, diverticulitis, and obstructive or perforated neoplasia. Patients who did not present at least 3 serum CRP levels within the first 7 postoperative days (PODs) were excluded. The patients were divided into 2 groups: leakage (n = 11) and non-leakage (n = 79). Clinical and demographic characteristics of the groups are shown in Table 1.

**Table 1:** Clinical and demographic characteristics.

Characteristic	Total (n = 90)	Non–leakage (n = 79)	Leakage (n = 11)	*P* value
Age, median (range), y	56.0 (36.2–68.0)	55.0 (37.5–67.0)	68.0 (36.0–77.5)	0.211
Gender, n (%)				1.000
Female	40 (44.4)	35 (44.3)	5 (45.5)	
Male	50 (55.6)	44 (55.7)	6 (54.5)	
Hospital stay, median (range), d	7.0 (7.0–9.0)	7.0 (7.0–7.5)	15.0 (10.5–22.0)	<0.001
Surgical indication, n (%)				0.182
Acute abdomen	23 (25.6)	23 (29.1)	0	
Colon adenocarcinoma	28 (28.9)	23 (29.1)	3 (27.3)	
Rectal adenocarcinoma	3 (3.3)	3 (3.8)	0	
Intestinal endometriosis	1 (1.1)	1 (1.3)	0	
Ostomy closure	33 (36.7)	26 (32.9)	7 (63.6)	
Colovesical fistula	1 (1.1)	1 (1.3)	0	
Megacolon	1 (1.1)	1 (1.3)	0	
Surgical procedure, n (%)				0.648
Right colectomy	25 (27.8)	23 (29.1)	2 (18.2)	
Left colectomy	3 (3.2)	3 (3.3)	0	
Segmental colectomy	4 (4.4)	3 (3.8)	1 (9.1)	
Transit reconstruction	33 (36.7)	27 (34.2)	6 (54.5)	
Rectosigmoidectomy	25 (27.8)	23 (29.1)	2 (18.2)	
Anastomosis, n (%)				0.292
Ileocolic	39 (43.3)	35 (44.3)	4 (36.4)	
Colorectal	42 (46.7)	39 (49.4)	3 (27.3)	
Colocolic	9 (10.0)	5 (6.3)	4 (36.4)	
Anastomosis technique, n (%)				0.059
Handsewn	20 (22.2)	15 (19.0)	5 (45.5)	
Stapled	70 (77.8)	64 (81.0)	6 (54.5)	
Surgical planning, n (%)				0.508
Emergency	26 (28.9)	24 (30.4)	2 (18.2)	
Elective	64 (71.1)	55 (69.6)	9 (81.8)	
Abdominal drain, n (%)				0.694
No	19 (21.1)	16 (20.3)	3 (27.3)	
Yes	71 (78.9)	63 (79.7)	8 (72.7)	
Death, n (%)				0.045
No	87 (96.7)	78 (98.7)	9 (81.8)	
Yes	3 (3.3)	1 (1.3)	2 (18.2)	

*P*-values obtained using the Mann–Whitney *U* test.

Serum CRP levels were evaluated on PODs 1 through 7 by immunoassays using the turbidimetric method with an Architect Plus C4000 analyser (Abbot, Lake Bluff, IL, USA). CRP levels >5 mg/L were considered altered. Patients were evaluated daily for the presence of abdominal pain, fever, volume, return of bowel habits, and/or appearance of abdominal drainage. Patients with altered parameters underwent laboratory and imaging examinations (CT or radiography). All patients received antibiotic prophylaxis, and mechanical preparation of the colon was conducted only for elective surgeries (71.1%).

Anastomotic leakage was defined using the following clinical and radiologic criteria: 1) presence of air or abscess near the site of anastomosis identified on CT, 2) purulent discharge or enteric secretion through the drain, and 3) clinical signs of peritonitis and/or presence of faecal or purulent discharge during surgical re-approach. Antibiotics were restarted in patients with leakage.

Categorical variables were presented as frequency and percentage, and quantitative variables, as median and interquartile range. The Shapiro–Wilk test was used to define normality, whereas the Mann–Whitney *U* test was used for bivariate comparisons. Receiver operating characteristic (ROC) curves were plotted using the values generated by logistic regression analysis. Sensitivity, specificity, negative predictive value (NPV), positive predictive value (PPV), accuracy, and area under the curve (AUC) were calculated. R language software (RStudio, Inc, Boston, MA, USA; www.rstudio.com) was used for statistical analysis with the level of significance set at 5% (*P* < 0.05).

### Ethical approval

The Research Ethics Committee of São Camilo University Centre approved this retrospective study by CAAE number: 66510317.6.0000.0062.

### Informed consent

The Research Ethics Committee of our institution approved this retrospective study and waived the need for informed consent.

## Results

During the study period, 90 patients underwent colorectal surgery with primary anastomosis. The median age was 56 years and 55.6% of patients were male (Table 1). Ostomy closure (36.7%) and colon adenocarcinoma resection (28.9%) were the most common surgical indications. The use of abdominal drainage did not affect the onset of leakage (*P* = 0.694). Colic anastomoses were created using a mechanical stapler in 70 patients (77.8%) and sutures in 20 patients (22.2%).

The postoperative mortality rate was significantly higher in patients with anastomotic leakage than in those without leakage (18.2% vs 1.3%; *P* < 0.045). The rate of postoperative complications was 26.6% (n = 24), including 11 patients (12.2%) in the leakage group: 10 (11.1%) with surgical site infection, two (2.2%) with evisceration, and one (1.1%) with pneumonia. Leakage was diagnosed between PODs 3 and 24 (median, 7.7 days) (Online Resource 1). The median hospital stay was significantly longer in the leakage group than in the non-leakage group (15 vs 7 days; *P* < 0.001). CT was used to diagnose anastomotic leakage in 72.7% of patients. Ten patients (91.9%) with leakage underwent surgical treatment, including four (36.4%) Hartmann’s colectomies, three (27.3%) colectomies with terminal ileostomy, three (27.3%) reanastomoses, and one (9.1%) abdominal drainage.

**Figure 1:**
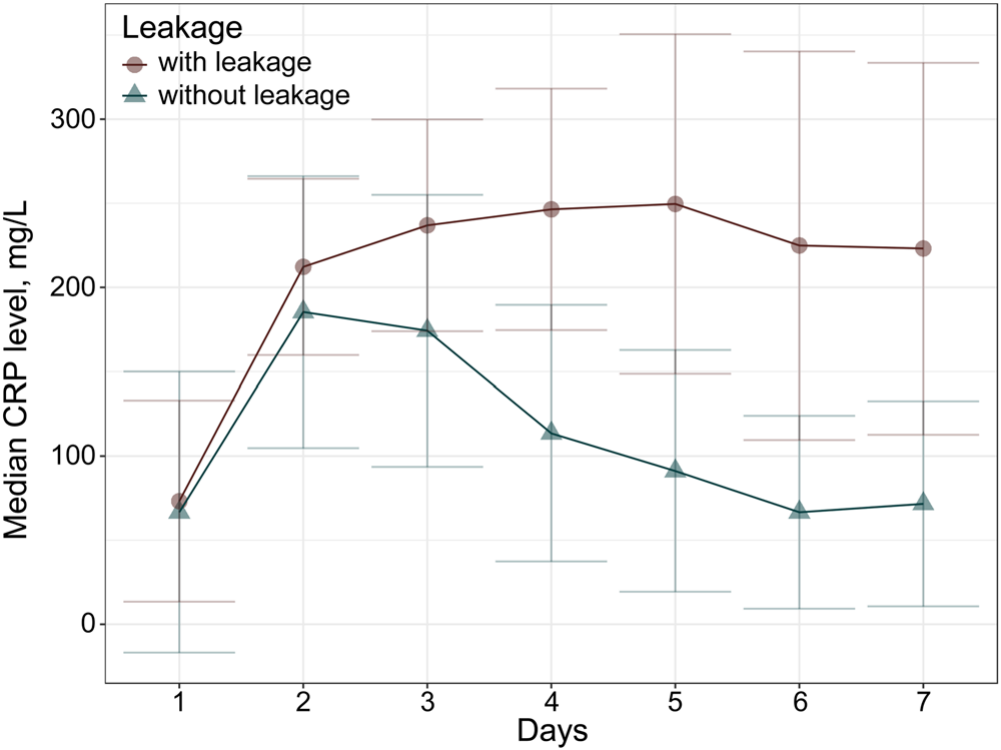
Serum C-reactive protein (CRP) levels in patients with and without primary colic anastomotic leakage.

There were no statistically significant differences in serum CRP levels in the first 3 PODs. After POD 4, however, there was a significant increase in serum CRP levels in patients with anastomotic leakage (median, 246.4 mg/L) compared with those without leakage (median, 113.5 mg/L; *P* = 0.002) (Online Resource 2). Serum CRP levels increased from POD 2 in patients with leakage and decreased in those without leakage (Fig. 1). Peak levels were seen on POD 5 in patients with leakage and on POD 2 in those without leakage.

**Figure 2:**
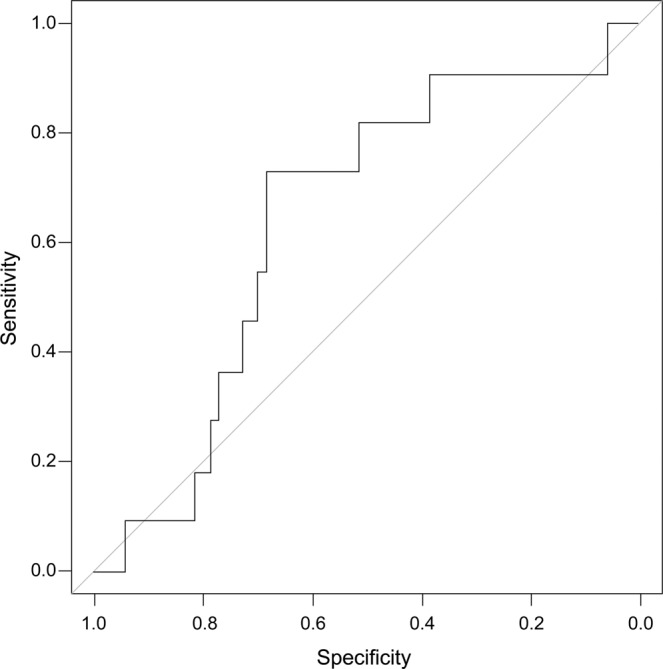
Receiver operating characteristic curve analysis of C-reactive protein level on postoperative day 3.

**Figure 3:**
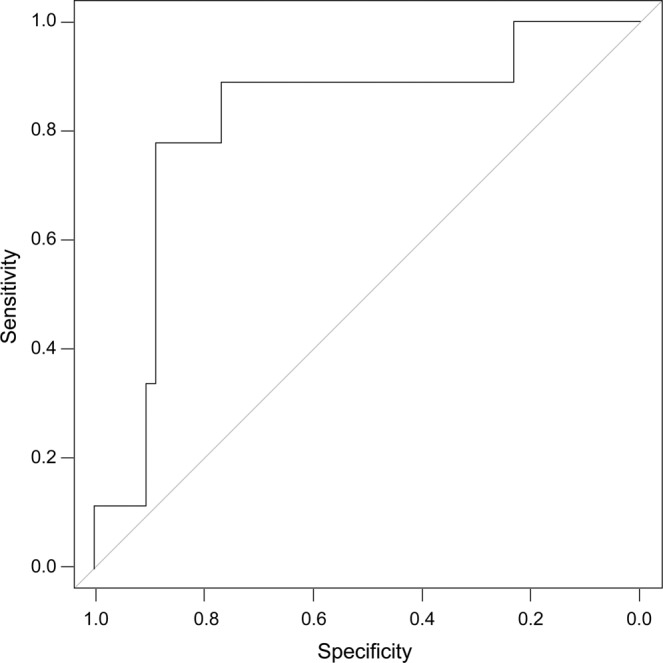
Receiver operating characteristic curve analysis of C-reactive protein level on postoperative day 4.

**Figure 4:**
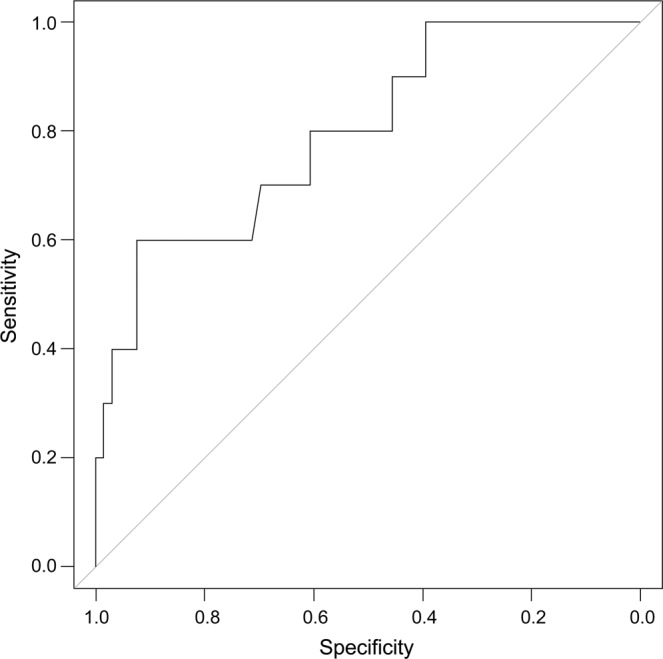
Receiver operating characteristic curve analysis of C-reactive protein level on postoperative day 5.

Analyses of the ROC curves from PODs 3 through 5 are presented in Tables 2–4. Sensitivity, specificity, NPV, PPV, accuracy, and AUC are presented in Figs. 2–4. A cutoff value of 220 mg/L was established on POD 3 with an AUC of 0.643, NPV of 89.3%, PPV of 20%, sensitivity of 71%, and specificity of 45%. On POD 4, with a cutoff value of 180 mg/L, the AUC was 0.821, NPV was 97.2%, sensitivity was 72.3%, and specificity was 88.9%. Patients with CRP levels <180 mg/L on POD 4 had a 12.2% probability of developing anastomotic leakage.

**Table 2:** Results of ROC curve analysis on POD 3 (cutoff value, 220 mg/L).

Accuracy	PPV	NPV	AUC	Sensitivity	Specificity
**0.679**	0.200	0.893	0.643	0.714	0.455

AUC, area under the curve; NPV, negative predictive value; POD, postoperative day; PPV, positive predictive value; ROC, receiver operating characteristic.

**Table 3:** Results of ROC curve analysis on POD 4 (cutoff value, 180 mg/L).

Accuracy	PPV	NPV	AUC	Sensitivity	Specificity
0.743	0.307	0.972	0.821	0.723	0.889

AUC, area under the curve; NPV, negative predictive value; POD, postoperative day; PPV, positive predictive value; ROC, receiver operating characteristic.

**Table 4:** Results of ROC curve analysis on POD 5 (cutoff value, 160 mg/L).

Accuracy	PPV	NPV	AUC	Sensitivity	Specificity
**0.789**	0.333	0.931	0.796	0.818	0.6

AUC, area under the curve; NPV, negative predictive value; POD, postoperative day; PPV, positive predictive value; ROC, receiver operating characteristic.

## Discussion

Anastomotic leakage is an undesirable complication of colorectal surgery^1^, resulting in increased length of hospitalization, increased treatment costs, delayed return of intestinal homeostasis, and decreased survival^34,35^. Because vital signs and leukocyte numbers are slow in responding, it is important to identify tools to detect early leakage^36,37^. Early diagnosis is essential to reduce morbidity and mortality^33,38–40^ because delayed diagnosis can increase mortality by 18%^37^.

CRP is an acute-phase protein produced by hepatocytes after inflammatory stimulation^18,25^. It is a useful marker to monitor and identify postoperative complications because it has a short half-life^38,40^. This protein has been shown to be as effective and sensitive as a predictor of anastomotic leakage^34,35,39–41^ and postoperative infection^15,19,38^. In fact, increased CRP levels are more sensitive to diagnose surgical complications than are increased erythrocyte sedimentation rate, leukocytes, body temperature, and heart rate^25^. Nevertheless, because of the individual regulation of inflammatory responses, disagreement among serum CRP levels is not uncommon^18,25^. Su’a *et al*. analysed 11 studies on anastomotic leakage and identified a wide variation in CRP cutoff values, ranging from 94 to 190 mg/L, on the same postoperative day^22^. Medications such as corticosteroids and statins may also alter this response, which could decrease serum CRP levels and alter the interpretation of cutoff levels^22^.

The surgical approach also influences serum CRP levels. Waterland *et al*. found higher CRP levels in patients who underwent open surgery than in those who underwent laparoscopic surgery^40^. They reported that a level of 123.5 mg/L on POD 4 after conventional surgery was the most predictive of anastomotic leakage. However, their study included only elective colorectal surgeries. Almeida *et al*. evaluated several types of colorectal resections and found that a CRP cutoff level of 140 mg/L on POD 3 had a significant association with the presence of anastomotic leakage^29^. Lagoutte *et al*. reported a cutoff value of 125 mg/L on POD 4^35^, whereas Granero-Garcia *et al*. reported that a cutoff level of 135 mg/L on POD 5 was a good predictor of leakage^39^. In another study, Muñoz *et al*. evaluated only patients who underwent elective laparoscopic colorectal cancer resection using the enhanced recovery after surgery (ERAS) protocol. In their study, CRP had a high NPV on POD 3 with a cutoff level of 163 mg/L^33^.

Singh *et al*. conducted a systematic review of 6 studies including >2400 patients^41^ and found that CRP levels were comparable in terms of accuracy on PODs 3, 4, and 5. On the other hand, Warschkow *et al*. conducted a meta-analysis and reported that CRP levels were more accurate on POD 4, demonstrating a high NPV for postoperative complications, with a cutoff value of 135 mg/L (38). Our study showed a high NPV, sensitivity, and specificity with a cutoff value of 180 mg/L on POD 4. This high cutoff value may be related to the inclusion of patients who underwent emergency colorectal surgery. This study also identified decreased CRP levels on POD 2 in patients without leakage, similar to the findings reported by Woeste *et al*.^34^. However, it is difficult to compare studies because of the non-standardization of anastomotic leakage definitions, day and time of CRP testing, patient selection, and surgical approach^22,34,41^.

Nonetheless, most studies support the notion that patients with anastomotic leakage present higher and sustained elevation of serum CRP levels in the postoperative period compared with patients without leakage^33,34,36,39,42^. According to several studies, increased serum CRP levels precede radiologic and clinical diagnosis of anastomotic leakage. They reported that the detection of sustained serum CRP elevation may decrease the time for indicating reoperation, which could lead to lower mortality rates and hospital costs^34,39^.

Sawyer *et al*. analysed the differences between short and extended use of antibiotics in >500 patients who underwent complicated intra-abdominal infection treatment and colorectal surgery. They found no significant differences in terms of surgical site infection, recurrent intra-abdominal infection, or death^43^. These findings suggest that with short-term use of effective and safe antibiotic therapy, patients undergoing emergency colorectal surgery could also benefit from the analysis of serial CRP levels, which could provide the possibility of early and safe hospital discharge^43^.

Patients tend to be discharged early, between PODs 4 and 5, with the advent of multimodal accelerated postoperative recovery protocols such as ERAS^33,35,41^. Because most surgical complications occur after patients are discharged, between PODs 5 and 8, a marker such as CRP, which has a high NPV on POD 4, could be used to exclude anastomotic leakage and other postoperative complications^38^. In addition to the use of scores to identify patients at high risk of anastomotic leakage, postoperative investigation protocols for patients with sustained elevation of CRP levels after POD 2 or with levels above the cutoff on POD 4 should be generated^13,38^. Because of the high NPV, serum CRP levels on POD 4 seem to play an important role in the exclusion of anastomotic leakage^33,35,39,40^.

In conclusion, serum CRP levels can be routinely analysed in patients who undergo elective or emergency colorectal surgery. Decreased CRP levels after POD 2 can exclude anastomotic leakage because they are not influenced by factors such as individual inflammatory response, type of approach, or surgical indication. A cutoff level of 180 mg/L on POD 4 can indicate high reliability for hospital discharge due to a low probability of anastomotic leakage.

Postoperative serum CRP levels in patients who undergo colorectal surgery with primary anastomosis could become a useful marker for the exclusion of anastomotic leakage. This was a single-centre study with a small sample size; therefore, prospective multicentre studies with a greater number of patients are necessary to confirm our findings and extend them to clinical practice.

## Supplementary information

Electronic Supplementary material.

## Data Availability

All data generated or analysed during this study are included in this published article.
