# Supplementary material for: Serum C-reactive protein is a useful marker to exclude anastomotic leakage after colorectal surgery
Source: Sci Rep. 2020 Feb 3;10:1687. doi: 10.1038/s41598-020-58780-3 (PMC6997159; doi:10.1038/s41598-020-58780-3)
Supplement: Supplementary file 1 — Electronic Supplementary material. [file 41598_2020_58780_MOESM1_ESM.docx]

**Electronic supplementary material for**

**Serum C-reactive protein is a useful marker to exclude anastomotic leakage after colorectal surgery**

**Journal: *Scientific Reports***

Bruno A Messias^a^; Ricardo V Botelho^b^; Sarhan S Saad^c^; Erica R Mocchetti^a^; Karine C Turke ^b,d^; Jaques Waisberg ^b,d^

^a^Department of Surgery, General Hospital of Carapicuiba, Carapicuiba, SP, Brazil

^b^Department of Surgery, State Public Servant Hospital (IAMSPE), São Paulo, SP, Brazil

^c^Department of Surgery, Paulista Medical School, Federal University of São Paulo, SP, Brazil

^d^Department of Surgery, ABC Medical School, Santo André, SP, Brazil

**Corresponding author:** Bruno A Messias

Department of Surgery, General Hospital of Carapicuiba, Carapicuiba, SP, Brazil.

72 Parkinson Avenue, Barueri- SP Zip code 06465-136, Brazil

Phone number +55 (11) 981745933

E-mail: [bruno22med@hotmail.com](mailto:bruno22med@hotmail.com)

**Short title:** CRP levels to exclude anastomotic leakage

**Online Resource 1:** Distribution of primary colic anastomotic leakage variables

| Variable, n (%) | Leakage (n = 11) |
| --- | --- |
| Leakage diagnosis |  |
| Abdominal drain | 3 (27.3) |
| Computed tomography | 8 (72.7) |
| Leakage treatment |  |
| Hartmann’s colectomy | 4 (36.4) |
| Colectomy and terminal ileostomy | 3 (27.3) |
| Colectomy and reanastomosis | 2 (18.2) |
| Laparotomy abdominal drainage | 1 (9.1) |
| Clinical | 1 (9.1) |
| Day of diagnosis |  |
| POD 3 | 1 (9.1) |
| POD 4 | 1 (9.1) |
| POD 5 | 2 (18.1) |
| POD 6 | 1 (9.1) |
| POD 7 | 3 (27.2) |
| POD 8 | 1 (9.1) |
| POD 9 | 1 (9.1) |
| POD 24 | 1 (9.1) |
| Median | 7.7 |

POD, postoperative day.

**Online Resource 2**. Serum CRP levels (median [range], mg/L)

| Timepoint | Total  (n = 90) | Non-leakage  (n = 79) | Leakage  (n = 11) | *P* value |
| --- | --- | --- | --- | --- |
| POD 1 | 70.1 (25.6-130.9) | 66.6 (24.1-138.8) | 73.2 (36.2-124.5) | 0.873 |
| POD 2 | 188.3 (126.2-246.8) | 185.4 (116.2-250.4) | 212.2 (200.2-220.3) | 0.813 |
| POD 3 | 183.4 (114.2-249.8) | 174.3 (110.8-248.5) | 236.9 (200.8-255.7) | 0.129 |
| POD 4 | 132.1 (72.5-200.1) | 113.5 (70.3-186.0) | 246.4 (220.9-250.2) | 0.002 |
| POD 5 | 96.7 (41.5-152.3) | 91.1 (40.0-136.8) | 249.6 (106.6-278.0) | 0.003 |
| POD 6 | 72.5 (39.2-130.6) | 66.6 (33.2-116.2) | 224.9 (62.8-286.8) | 0.012 |
| POD 7 | 87.9 (51.2-166.4) | 71.6 (52.9-133.9) | 223.1 (126.7-246.3) | 0.079 |

*P* values obtained using the Mann-Whitney *U* test. CRP, C-reactive protein; POD, postoperative day.
